# Influence of Hypochlorous Acid Obtained From an Electrolytic Device on the Mechanical Properties of Root Dentin

**DOI:** 10.1002/cre2.70332

**Published:** 2026-04-28

**Authors:** Matheus Albino Souza, Carolina Bianchi Farina, Anna Vithoria da Costa Longhi, Felipe Gomes Dallepiane, Camila Yasmin Monteiro Pizzi, Nathan Mateus Piccolo, Ana Beatriz Canabarro Zart, Bianca Gonçalves Trindade, Leonardo Scarparo Rissardo, Natália Ulmi Ziglioli, Vicenzo Ghisleni Arenhardt, Liviu Steier, José Antônio Poli de Figueiredo

**Affiliations:** ^1^ School of Dentistry University of Passo Fundo Passo Fundo Brazil; ^2^ School of Dental Medicine University of Pennsylvania Philadelphia Pennsylvania USA; ^3^ School of Dentistry Federal University of Rio Grande do Sul Porto Alegre Brazil

**Keywords:** hypochlorous acid, mechanical properties, root dentin, sodium hypochlorite

## Abstract

**Objectives:**

Sodium hypochlorite (NaOCl) exhibits antimicrobial activity. However, it is associated with negative effects in endodontics, such as cytotoxicity and alterations in the mechanical properties of root dentin, leading to the search for new alternatives. Hypochlorous acid (HClO) exhibits antimicrobial activity and low cytotoxicity as irrigation solution. Therefore, the aim of this study was to evaluate the influence of HClO on the mechanical properties of root dentin.

**Material and Methods:**

Samples were obtained from 120 mandibular bovine incisors, which were distributed into four experimental groups: microhardness (30 teeth/60 samples), flexural strength (15 teeth/60 samples), cohesive strength (15 teeth/60 samples), and fracture resistance (60 teeth/60 samples). For all tests, the samples were subdivided into six groups (*n* = 10): G1: distilled water (DW); G2:1% NaOCl; G3:2.5% NaOCl; G4:5.25% NaOCl; G5:250 ppm HClO; and G6:500 ppm HClO. The irrigation protocol was set at 30 min. Then, a Vickers tester was used to evaluate microhardness, and flexural strength, cohesive strength, and fracture resistance were evaluated in a universal testing machine. One‐way ANOVA and Tukey tests were used for multiple comparisons in all evaluations (*α* = 5%).

**Results:**

The highest microhardness was observed in DW, 250 ppm HClO and 500 ppm HClO, with no statistical differences between them (*p* > 0.05). The highest flexural and cohesive strength were observed in DW, 1% NaOCl, HClO 250 ppm, and HClO 500 ppm, with no statistical differences between them (*p* > 0.05). Regarding the fracture resistance, there were no statistical differences between all groups (*p* > 0.05).

**Conclusion:**

It was concluded that HClO preserved the mechanical properties of root dentin.

## 1 Introduction

During instrumentation, the use of irrigation solutions with recognized antimicrobial potential is essential, considering the importance of microorganisms in the etiology of pulpal and periapical changes. In this sense, the main objective is to promote an effective decontamination of the root canal system, creating favorable conditions for the repair and health of the periapical tissues (Gomes et al. 2023). At the same time, the preservation of the dentin structure is extremely important in this process, taking care to ensure that the use of these irrigant solutions does not promote significant changes and compromise the longevity of the endodontically treated tooth (Marending et al. 2007).

The sodium hypochlorite (NaOCl) has been used as irrigation solution in endodontics, once it presents broad‐spectrum antimicrobial action (Estrela et al. 2002) and promotes the chemical removal of structured microbial biofilm (Busanello et al. 2019). Moreover, it has the ability to dissolve organic tissue (Okino et al. 2004). However, the use of NaOCl has been related to negative effects in endodontic therapy, such as cytotoxicity (Gatot et al. 1991) and changes in the mechanical properties of root dentin (Moreira et al. 2009). Its cytotoxicity can induce inflammatory events when NaOCl is extruded to periapical region (Gatot et al. 1991), while changes in the dentin structure can induce root fracture (Moreira et al. 2009). Therefore, these negative effects lead to the search for new alternatives in the field of irrigation solutions in endodontics.

Hypochlorous acid (HClO) is recognized for its antimicrobial properties, inducing the irreversible oxidation of sulfhydryl groups of bacterial enzymes, interrupting the bacterial cell metabolic reactions and causing damage to DNA of bacterial cell (Baumgartner and Ibay 1987). More recently, the Dentaqua device (Dentaqua, Conmel, Ireland) was developed, being able to produce a stable hypochlorous HClO solution at different concentrations. It is obtained by the electrolysis of saline solution mixed with distilled water. According to previous studies, HClO presented high antimicrobial activity in root canals infected with *Enterococcus faecalis* and low cytotoxicity over fibroblastic cells (Souza, Steier et al. 2024; Souza, Zanella 2024). Despite these promising results, there are no studies in the literature revealing the impact of HClO solution in the mechanical properties of root dentin.

Thus, this study aimed to evaluate the influence of HClO solution obtained from an innovative electrolytic device on the mechanical properties of the root dentin. The null hypothesis was that (i) HClO does not induce any significant modifications in the mechanical properties of root dentin.

## 2 Material and Methods

### 2.1 Sample Obtaining and Preparation

This study did not require approval from the local Ethics Committee for the Use of Animals, as it uses bovine teeth obtained from animals slaughtered for commercial purposes, where the teeth would be discarded. For this study, 120 extracted lower bovine incisors were distributed into four test groups: microhardness (30 teeth), flexural strength (15 teeth), cohesive strength (15 teeth), and fracture resistance (60 teeth). The sample calculation was performed using the 3.1 version of G*Power software (Heinrich Heine, Universität Düsseldorf), selecting the Student's *t*‐test. An alpha error of 0.05 and a beta power 0.95 were also stipulated. A total of eight samples per group was indicated as the ideal size required for non‐significant differences. An additional two specimens per group were used to compensate for possible losses.

All selected teeth were intact, with straight roots and formed root apex. After extraction, teeth were cleaned with periodontal instruments, immersed in 0.9% sterile saline solution, and stored under refrigeration at 1°C temperature for a maximum period of 3 months before use. Dental crowns were sectioned with a diamond disc (KG Sorensen, Serra, ES, Brazil), obtaining roots 15 mm in length. The coronal portion was sectioned from the cementoenamel junction and two markings were made to standardize the roots, one 5 mm and the other one 10 mm above the apex.

All roots were prepared to remove pulp tissues and to standardize the canal diameter. Working length (WL) was established by introducing #10 K‐file (Dentsply‐Sirona, York, PA, USA) into the root canal until its tip was visualized at the apical foramen. From this measure, 1 mm was subtracted from WL. Roots were enlarged using manual K‐files (Dentsply‐Sirona) and serial instrumentation, up to a #45 file. Distilled water (DW) (Natupharma, Passo Fundo, RS, Brazil) was used as an irrigation solution and renewed at each instrument change. The root canals were fully filled with DW, the instrumentation was performed with each instrument and irrigation with 5 mL of DW was performed after the use of each instrument, totaling a volume of around 30 mL of DW during the instrumentation. After that, the root canals were irrigated with 3 mL of 17% EDTA (Maquira, Maringá, PR, Brazil) for 1 min, in order to remove the smear layer, followed by irrigation with 5 mL of DW and drying with absorbent paper points (Tanari, Manacapuru, AM, Brazil).

### 2.2 Irrigation Solutions

The pure DW was obtained by a compounding pharmacy (Natupharma, Passo Fundo, RS, Brazil). In the same way, the NaOCl solutions at concentrations of 1%, 2.5%, and 5.25% were obtained by a compounding pharmacy (Natupharma, Passo Fundo, RS, Brazil), which carried out the titration of the aforementioned concentrations.

**Figure 1:**
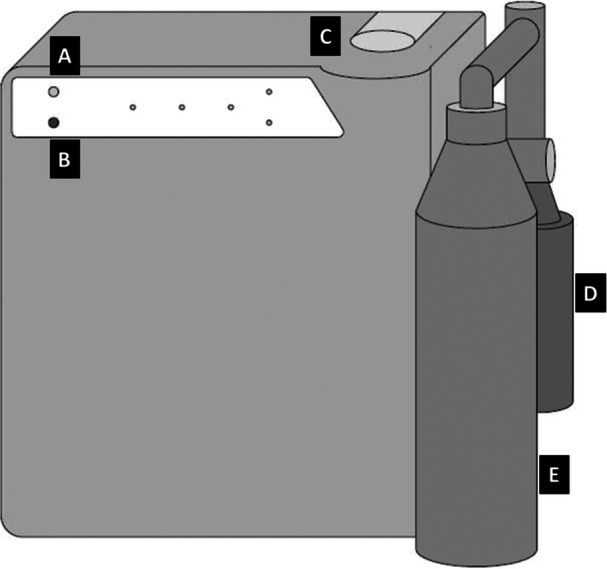
Representative image of the Dentaqua device. A—water bottle button; B—ecasol button; C—upper compartment; D—smaller bottle with brine solution; E—larger bottle with HClO solution.

**Figure 2:**
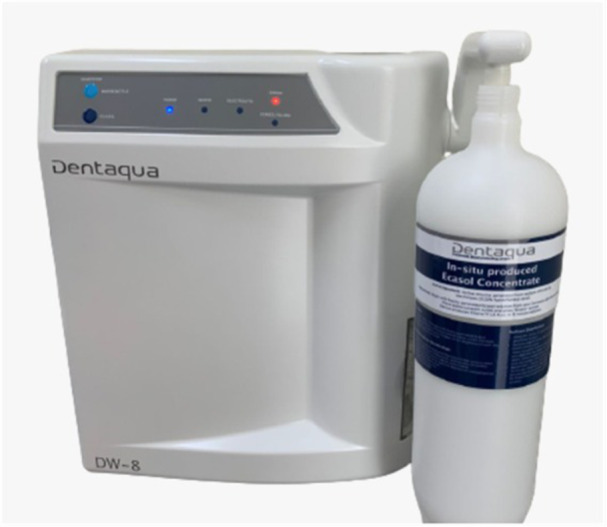
Image of the Dentaqua device.

The 250 and 500 ppm HClO solutions were obtained by a specific Dentaqua device (Dentaqua, Conmel, Ireland), for each concentration. This device consists of a compartment on the top, a connector for the smaller bottle on the back, and a connector for the larger bottle on the front. Figure 1 provides an illustration and Figure 2 provides a real image of the Dentaqua device. The electrochemical activation technology involves the generation of electrochemically activated solutions by passing a dilute NaCl solution through an electric field in a flow‐through electrolytic module, segregating the ions formed and producing two oppositely charged solutions with altered physical and chemical properties. The positively charged solution (anolyte) consists of a mixture of unstable mixed oxidants, such as HClO, in a physically excited state, which is capable of penetrating biofilms and is highly microbicidal. The negatively charged antioxidant solution (catholyte) predominantly consists of sodium hydroxide in an excited state.

**Figure 3:**
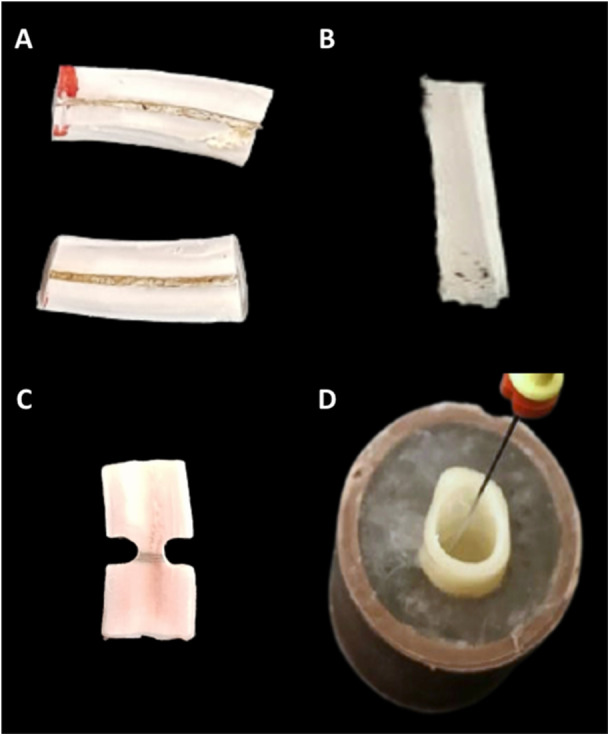
Illustrative images of samples prepared for evaluation of A—microhardness; B—flexural strength; C—cohesive strength; and D—fracture resistance.

The upper compartment is filled with sterile DW. The smaller bottle on the back is filled with brine solution at concentration of 9%, which is composed of sodium chloride. After filling, the smaller bottle was connected to the back of the device, while the larger bottle was connected to the front of the device. The Water Bottle button was pressed, performing a washing cycle of the device with DW for 3 min, discarding this solution in the larger bottle. The DW was then discarded and the larger bottle was again connected to the device for the production of HClO. The Ecasol button was pressed for the production of HClO for 10 min, which was obtained by the electrolysis of saline solution mixed with DW in the device, dispensing the HClO solution at 250 ppm in the larger bottle, being ready to use. The production of HClO solution at 500 ppm occurs similarly since engineering adjustments within the device performed by the manufacturer provided the production in a higher concentration.The Figure 3 provides an illustration of the samples prepared for the tests that will be described below.

### 2.3 Microhardness

Thirty bovine roots were used for this evaluation. Two longitudinal grooves were performed on the external root surface using a diamond disc (KG Sorensen, Serra, ES, Brazil), being careful to preserve the root canal space. Roots were split into two halves with microtome blade, obtaining 60 samples. The samples were fixed in acrylic resin blocks, leaving the root dentin exposed upwards. The polishing was performed with silicon carbide abrasive paper, following the sequence 180, 320, and 600 grit (3M ESPE, St. Paul, MN, USA), and 0.25‐mm diamond polishing paper (Metkon, Bursa, Turkey), under cooling with DW. After this preparation, the samples were washed with DW and dried with aspiration cannula.

The 60 samples were divided into six groups (*n* = 10), according to the tested irrigation protocols: G1: DW (control); G2: 1% NaOCl; G3: 2.5% NaOCl; G4: 5.25% NaOCl; G5: HClO 250 ppm; G6: HClO 500 ppm. Each sample was placed in plastic tubes (Thermo Fischer Scientific – São Paulo, SP, Brazil) and completely covered with 3 mL of the tested irrigation solution. From the moment the sample was completely covered, 2 mL of the tested irrigation solution was added, in order to ensure that each sample was completely wetted with the experimental agents inside the plastic tube. Then, the tested irrigation solution remained in contact with the samples for 5 min. Subsequently, irrigation with 5 mL of the tested irrigation solution was performed and the tested irrigation solution was renewed. Six 5‐min irrigation cycles were performed, totaling 30 min of contact with each sample. Finally, irrigation with 5 mL of DW was performed and the samples of all groups were dried using aspiration cannula (Golgran, São Caetano do Sul, SP, Brazil).

After the tested irrigation protocols, the microhardnes was measured in each sample by using a Durascan 20 Vickers tester (HMV‐2000, Shimadzu, Kyoto, Japão) at 40× magnification, 300‐g load, and 20‐s dwell time. Three indentations were performed along parallel lines to the edge of the root canal lumen, being the first one at 1.000 μm from the root canal entrance, and two others at a distance of 200 μm from each other. The microhardness value for each sample was obtained as the average value obtained for the three indentations.

### 2.4 Flexural Strength

Fifteen bovine roots were used for this evaluation. After sample preparation, the roots were sectioned along the long axis and polished using abrasive papers with 500, 800, 1000, and 1200 grit (3M ESPE, St. Paul, MN, USA). It was obtained 60 dentin sticks in a rectangular format, with the following standardization: 0.18 mm in thickness × 1.8 mm in width × 5.0 mm in length. The 60 dentin sticks were distributed in the same six previously described groups (*n* = 10) and submitted to the same irrigation protocols, as previously described in the microhardness evaluation.

The flexural strength test was performed using a miniature three‐point device with a 3 mm support extension. Each stick was inserted into the support span. The load was applied to the central portion of the stick using a universal testing machine (EMIC DL 2000 – São José dos Pinhais, PR, Brazil) at a speed of 0.5 mm/min. The load was applied until the point that fracture occurred. The flexural strength of each sample was calculated using the formula: 3 PL/2bh^2^, where *P* is the maximum load until the moment of fracture (N), *L* is the distance between support points (mm), *b* is the sample width (mm), and *h* is the sample height (mm). The data were expressed in MPa.

### 2.5 Cohesive Strength

Fifteen bovine roots were used for this evaluation. After the sample preparation, the roots were sectioned along the long axis and a perpendicular cut to the long axis was performed on both halves, producing 4 fragments of root dentin. Specimens were manually cut into an hourglass shape using a cylindrical drill with a cross‐sectional area of approximately 0.8 mm^2^. Then, 60 hourglass‐shaped dentin samples were obtained, being distributed in the same six previously described groups (*n* = 10), and submitted to the same irrigation protocols, as previously described in the microhardness evaluation.

Then, each sample was fixed on a microtensile test loop with cyanoacrylate (Loctite Super Bonder; Henkel Loctite Corporation, Rocky Hill, CT, USA) and submitted to a cohesive load at 0.5 mm/min in a universal testing machine (EMIC DL 2000, São José dos Pinhais, PR, Brazil). The cross‐sectional area at the fracture site was measured with a digital caliper and the cohesive strength was calculated using the following formula: *σ* = F/A, where *σ* represents the maximum cohesive strength, *F* (N) represents the force used, and *A* (mm^2^) represents the area of the fracture site. The data were expressed in megapascals (MPa).

### 2.6 Fracture Resistance

Sixty extracted bovine teeth were used for this evaluation. After sample preparation, reference markings were performed on root surfaces using an overhead projector pen, whose location was obtained using a digital pachymeter ruler. The first mark was performed 2 mm below the uppercut, the second 2 mm above the root apex, and the third mark at mid‐distance between the two marks. The buccolingual diameter was 6.5 mm in the cervical third, 5.5 mm in the middle third, and 4 mm in the apical third, and the mesio‐distal diameter was 5.0 mm in the cervical third, 4.5 mm in the middle third, and 3.5 mm in the apical third. Root canals were scaled with the aid of a cylindrical diamond bur No. 1090 (KG Sorensen, São Paulo, SP, Brazil). During this procedure, constant measurements were performed with the digital pachymeter ruler (Mitutoyo Jundiaí, SP, Brazil) adapted for internal canals, until dentin walls of 1 mm in thickness were obtained in all roots. DW was used to remove remnants from the root canal preparation.

Roots were embedded in acrylic resin using plastic cylindrical PVC molds of 1⁄2 inch in thickness and 20 mm in length (Tigre do Brasil, Osasco, SP, Brazil), maintaining root exposure of 3 mm to simulate the biological space. The 60 roots were distributed in the same six previously described groups (*n* = 10). In all groups, the root canals were fully filled with the tested irrigation solution using a 5‐mL syringe (BD, Curitiba, PR, Brazil) with a 19‐G needle (Ultradent, Indaiatuba, SP, Brazil). The tested irrigation solution remained in contact with root canal walls for 5 min. Subsequently, irrigation with 5 mL of the tested irrigation solution was performed and the tested irrigation solution was renewed. Six 5‐min irrigation cycles were performed, totaling 30 min of the tested irrigation solution into the root canals. Finally, irrigation with 5 mL of DW was performed and the root canals of all groups were dried using an aspiration cannula (Golgran, São Caetano do Sul, SP, Brazil) and absorbent paper points (Tanari, Manacapuru, AM, Brazil).

Then, specimens were positioned in the lower part of the universal testing machine (EMIC DL 2000, São José dos Pinhais, PR, Brazil) and a compressive load was vertically applied to the coronal surface of roots. The loading speed of 1 mm/min was used until the record of root fracture. The load at which the fracture occurred was recorded and expressed in Newtons (N).

### 2.7 Statistical Analysis

Data were analyzed using SPSS 11.0 software (SPSS, Chicago, IL, United States) in all experimental tests. The Shapiro–Wilk test confirmed the normal distribution of the data. Data from all evaluations were analyzed by one‐way ANOVA followed by post‐hoc Tukey's test (*p* < 0.05).

## 3 Results

The mean (standard deviation) of Vickers microhardness values, flexural strength (MPa), cohesive strength (MPa), and fracture resistance of root dentin after tested irrigation protocols are presented in Table 1. The highest microhardness values were observed in DW, HClO 250 ppm and HClO 500 ppm groups, being statistically different from all other groups (*p* < 0.05), with no statistically significant differences between them (*p* > 0.05). The lowest microhardness values were observed in 5.25% NaOCl group, being statistically different from all other groups (*p* < 0.05). The highest flexural strength and cohesive strength values were observed in DW, 1% NaOCl, HClO 250 ppm, and HClO 500 ppm groups, with no statistically significant differences between them (*p* > 0.05). The lowest flexural strength and cohesive strength values were observed in 2.5% NaOCl and 5.25% NaOCl groups, with no statistically significant differences between them (*p* > 0.05). Regarding fracture resistance, there were no statistically significant differences between all groups (*p* > 0.05).

**Table 1:** Mean (standard deviation) of Vickers microhardness values, flexural strength (MPa), cohesive strength (MPa), and fracture resistance (N) of root dentin after tested irrigation protocols.

Group	Microhardness values	Flexural strength	Cohesive strength	Fracture resistance
1. DW	43.21 (3.84)^a^	9.26 (1.54)^a^	53.23 (4.89)^a^	257.26 (40.02)^a^
2. NaOCl 1%	34.17 (1.12)^b^	8.74 (1.16)^a^	54.32 (5.98)^a^	302.55 (39.70)^a^
3. NaOCl 2.5%	30.05 (2.72)^c^	5.57 (0.87)^b^	39.23 (2.71)^b^	313.41 (42.48)^a^
4. NaOCl 5.25%	25.64 (0.89)^d^	4.94 (1.03)^b^	38.60 (2.82)^b^	258.29 (37.14)^a^
5. HClO 250 ppm	39.29 (1.91)^a^	9.33 (1.79)^a^	46.20 (4.17)^a^	327.68 (34.96)^a^
6. HClO 500 ppm	39.98 (2.86)^a^	8.16 (1.08)^a^	48.53 (3.45)^a^	257.63 (31.64)^a^

*Note:* Different superscript letters (a–d), in the column, indicate significant differences between groups (*p* < 0.05).

Abbreviations: DW, distilled water; HClO, hypochlorous acid; NaOCl, sodium hypochlorite.

## 4 Discussion

The present study was designed to compare the effects of tested irrigation solutions on the mechanical properties of root dentin, such as microhardness, flexural strength, cohesive strength, and fracture resistance. These properties are clinically relevant as they act as indicators of mineral loss or gain in the dental hard tissues and amount of force required for the failure of mechanical bonds in the root dentin (Sim et al. 2001; Zaparolli et al. 2012). As a consequence, the alteration in some level of these properties after root canal therapy may predispose the root fracture, leading to extraction of endodontic treated teeth. It is known that NaOCl solutions impair the mechanical properties of root dentin (Moreira et al. 2009) and promote degradation of dentin collagen (OYARZUN et al. 2002). In the present study, HClO obtained from an electrolytic device was evaluated at concentrations of 250 and 500 ppm, making a comparison with several concentrations of NaOCl. Since HClO presented satisfactory antimicrobial action and low cytotoxicity (Souza, Steier et al. 2024; Souza, Zanella 2024), and there are no studies in the literature regarding the influence of HClO on dentin structure, the present study evaluated its effects on the mechanical properties of root dentin.

Bovine teeth were used in the present study, due to the ease of obtaining and the similarity with human root dentin, considering the number and diameter of dentinal tubules (Schilke et al. 2000). In addition, the bovine teeth are obtained from animals slaughtered with standardized age, genetic lineage, and diet. It ensures greater homogeneity of the structure and mineral composition of the root dentin (Wegehaupt et al. 2010). Regarding the tested irrigation solutions, NaOCl and HClO represent chlorine‐containing solutions. However, they have different concentrations and pH, knowing that pH is reflective of the chlorine forms available in these solutions (Saleh and Ettman 1999). It is also well known that the concentration of NaOCl solutions has influence in the effects over the dentin structure (Ghisi et al. 2015). For these reasons, several concentrations of NaOCl were tested in the present study. In the same way, the HClO was evaluated at concentrations of 250 and 500 ppm. These are the concentrations that the Dentaqua device can produce at the time of use, being a difference in relation to conventional forms of HClO that are commercially available or those produced by previous equipments. It minimizes the variables of chemical instability and/or loss of concentration over time or storage. Finally, the irrigation cycles were performed, with constant renewal of the tested irrigation solutions, totaling 30 min of contact with the dentin structure, in order to simulate the clinical reality of the time required to perform the chemo‐mechanical preparation in all its stages (Siqueira and Rôças 2008).

The Vickers tester represents one of the most usual devices for microhardness evaluation in the root dentin, providing an effective measurement range and sensitivity when equal loads are applied to the dentin surface (Cruz‐Filho et al. 2011). For these reasons, it was used in the present study. Another topic refers to the need of microhardness measurement in two moments, being before and after the tested protocols. However, the literature shows that there is no significant difference in microhardness values when the measurement was carried out at these two moments in a group treated with DW. Therefore, the microhardness was measured after the tested irrigation protocols, making a comparison with the control group, which was treated with DW. The evaluation of other mechanical properties was performed by using the universal testing machine. It is a commonly used method, compatible with the clinical situation and induces less stress on the dentin structure (de Andrade Marafiga et al. 2022).

According to the results of the present study, the use of HClO at concentrations of 250 and 500 ppm resulted in higher values of root dentin microhardness, indicating a preservation of this mechanical property. Even though HClO contains chlorine in its composition, this concentration is lower when compared to NaOCl solutions, resulting in a lower impact on the dentin structure. At the same time, the present study revealed that NaOCl solution decreased the microhardness values in a significant way, even when used at a low concentration of 1%. The impact was even greater when NaOCl was used at a high concentration of 5.25%. Similar findings were found in previous studies, where NaOCl significantly reduced the microhardness of root dentin (Cecchin et al. 2017; Slutzky‐Goldberg et al. 2004; Aslantas et al. 2014). The NaOCl promotes deterioration of the organic component of dentin, formed mainly by collagen, causing mechanical changes and reduction of root dentin microhardness. Clinically, it would result in a more brittle and less resistant substrate, propagating fatigue cracks during cyclic stresses and increasing the possibility of root fracture (Kruzic and Ritchie 2008).

The reduction of flexural strength decreases the stress that is required to cause fracture of dentin structure (Cullen et al. 2015). In turn, the reduction of cohesive strength compromises the elasticity modulus, as well as the connective tissue of the root dentin (Sayin et al. 2009). According to previous studies, the use of NaOCl at high concentrations induces a negative impact on the flexural strength (Cecchin et al. 2015) and the cohesive strength (Cecchin et al. 2017) of root dentin. Similar findings were found in the present study, where the use of NaOCl at 2.5% to 5.25% decreased the flexural and cohesive strength of root dentin, inducing a higher impact on the mechanical properties when compared to 1% NaOCl. Due to decalcification effects in the inorganic components and deleterious effects in the collagen provided by NaOCl at high concentrations, it led to an increased ratio of apatite crystallites compared with collagen, making the dentin more brittle. In addition, it was found that deleterious effects over flexural and cohesive strength of root dentin are dependent of concentration of NaOCl solutions (Sayin et al. 2009). It helps to explain the reasons for low concentration of 1% NaOCl and both concentrations of HClO preserve these mechanical properties in the present study, considering a lower amount of active chlorine in the composition of these auxiliary chemical substances.

Some factors predispose the endodontically treated teeth to be more susceptible to fracture, such as dental structure loss and altered mechanical properties of root dentin. In addition, the use of different irrigation solutions can compromise the fracture resistance of root dentin (Cecchin et al. 2015). Although the use of NaOCl solutions resulted in a reduction in the values of microhardness, flexural strength, and cohesive strength of root dentin, no significant differences were observed between the control groups and the groups treated with NaOCl and HClO in different concentrations, when fracture resistance was evaluated in the present study. During the microhardness, flexural strength and cohesive strength evaluation, the samples were completely immersed in the tested solutions. It ensures a much larger volume of auxiliary chemical substance in contact with the root dentin, when compared to the samples of fracture resistance evaluation. In addition, static loading was applied in the present study, generating forces which exceed the physiological range of forces that teeth endure during everyday activities, such as chewing and biting. These higher forces used in the present study are more representative of those seen in traumatic tooth fracture, rather than from everyday tooth function. Thus, specimens could have been subjected to cyclic loading followed by static loading, and finally thermocycling, to mimic the clinical scenario (Ordinola‐Zapata et al. 2022). On this way, more significant differences could be found in the fracture resistance evaluation, being a limitation of present study. Anyway, the groups treated with HClO preserved the fracture resistance of root dentin, in the same way as observed in the other evaluations of the present study.

The present study proposes the use of Dentaqua electrolytic device, in order to produce a pure and fresh solution containing HClO. It ensures obtaining of a stable irrigation solution, with no risk of concentration reduction through storage. According to the results of the present study, HClO at concentrations of 250 and 500 ppm did not promote any significant modifications in the mechanical properties of root dentin, confirming the null hypothesis of the present study. Previously, HClO solutions revealed effective antimicrobial action and induction of low levels of cytotoxicity, when used at concentrations of 250 and 500 ppm (Souza, Steier et al. 2024; Souza, Zanella 2024). Given the above and considering the imminent possibility of additional studies, HClO obtained from the Dentaqua electrolytic device may become an alternative irrigation solution to be used during endodontic treatment. Additional studies are needed, in order to observe the effects of the HClO in scenarios that simulate in a better way the clinical and functional reality, such as thermocycling studies, microscopic evaluations and in vivo studies.

## 5 Conclusion

Under the study limitations, it was possible to conclude that HClO in both tested concentrations preserved the mechanical properties of the root dentin.

## Author Contributions

**Matheus Albino Souza:** supervision, article writing, statistical analysis, article review. **Carolina Bianchi Farina:** article writing, methodology. **Anna Vithoria da Costa Longhi:** article writing, statistical analysis, article review. **Felipe Gomes Dallepiane**, **Camila Yasmin Monteiro Pizzi**, **Nathan Mateus Piccolo**, **Ana Beatriz Canabarro Zart**, **Bianca Gonçalves Trindade**, **Leonardo Scarparo Rissardo**, **Natália Ulmi Ziglioli**, and **Vicenzo Ghisleni Arenhardt:** methodology. **Liviu Steier** and **José Antônio Poli de Figueiredo:** supervision, article writing, statistical analysis, article review.

## Funding

The authors received no specific funding for this work.

## Ethics Statement

This study did not require approval from the research ethics committee, as it uses bovine teeth obtained from animals slaughtered for commercial purposes.

## Consent

Informed consent is not applicable due to the fact that samples were obtained from animals slaughtered for commercial purposes.

## Conflicts of Interest

The last but one author owns IP rights on the Dentaqua. The rest of the authors declare no conflicts of interest. The rest of the authors have no financial affiliation (e.g., employment, direct payment, stock holdings, retainers, consultantships, patent licensing arrangements, or honoraria) or involvement with any commercial organization with a direct financial interest in the subject or materials discussed in this manuscript, nor have any such arrangements existed in the past 3 years. The other authors declare no conflicts of interest.

## Data Availability

The authors have nothing to report.
